# Immunoglobulin G; structure and functional implications of different subclass modifications in initiation and resolution of allergy

**DOI:** 10.1002/iid3.192

**Published:** 2017-11-21

**Authors:** Timothy H. Scott‐Taylor, Stefan‐Claudiu Axinia, Sumeya Amin, Ruth Pettengell

**Affiliations:** ^1^ School of Life Sciences London Metropolitan University 166‐220 Holloway Road London, N7 8DB; ^2^ Department of Haematology St George's University of London Cranmer Terrace London SW17 0RE

**Keywords:** Allergy, antibodies, cells, mast cells/basophils, molecules, processes

## Abstract

IgE and not IgG is usually associated with allergy. IgE lodged on mast cells in skin or gut and basophils in the blood allows for the prolonged duration of allergy through the persistent expression of high affinity IgE receptors. However, many allergic reactions are not dependent on IgE and are generated in the absence of allergen specific and even total IgE. Instead, IgG plasma cells are involved in induction of, and for much of the pathogenesis of, allergic diseases. The pattern of IgG producing plasma cells in atopic children and the tendency for direct or further class switching to IgE are the principle factors responsible for long‐lasting sensitization of mast cells in allergic children. Indirect class switching from IgG producing plasma cells has been shown to be the predominant pathway for production of IgE while a Th2 microenvironment, genetic predisposition, and the concentration and nature of allergens together act on IgG plasma cells in the atopic tendency to undergo further immunoglobulin gene recombination. The seminal involvement of IgG in allergy is further indicated by the principal role of IgG4 in the natural resolution of allergy and as the favourable immunological response to immunotherapy. This paper will look at allergy through the role of different antibodies than IgE and give current knowledge of the nature and role of IgG antibodies in the start, maintenance and resolution of allergy.

## Introduction

IgE and not IgG is usually associated with allergy. Allergic sensitization is conventionally thought of as the establishment of a population of IgE making plasma cells which induce the degranulation of vasoactive amines from gut, skin, or lung mast cells and blood basophils and cationic proteins from eosinophils. IgE lodged on mast cells and basophils through the prolonged expression of high affinity IgE receptors allows for the protracted duration of allergic. But the initial allergic symptoms may originate from the pathogenesis of IgG producing B cell clones to allergens. The frequency of B cells in allergic children switching from IgG to IgE production may be the critical difference in atopic children that enables priming of mast cells and basophils.

However, many allergic reactions are not dependent on IgE and are generated in the absence of allergen specific and even total IgE. Instead, IgG plasma cells are involved in induction of allergy. It has been shown lately that B cells start secreting IgG but are disposed in allergic people either by an inherent genetic disposition or experience in the womb or soon after birth, resulting in a Th2 polarized immune reaction to allergenic compounds that generates class switching of initially IgG secreting B cells to produce IgE, thereby potentiating an allergic or anaphylactic reaction. Additionally, IgG may be involved in the resolution of allergy. IgG4, in particular, is produced or correlates with desensitization of allergy by immunotherapy. The structure of IgG4 enables the blockading of IgG receptors and, because IgG4 is unstable and mops up antigen in blood as a monovalent protein, there is reduced free allergen to stimulate IgE on sensitized mast cells and basophils. IgG4 also reacts with FcγIIb, the inhibitory immunoglobulin receptor present on monocytes, macrophages, and dendritic cells and reduced allergic reactions through the production of IL‐10. Thus, the induction, the pathology or the resolution of allergy entail other factors than specific IgE to allergens. Allergic reactions involve a large number of factors and the formation of allergic conditions involves IgG in the establishment of atopy, the generation of clinical symptoms of allergy and in the amelioration of the response and resolution of allergy. The pattern of IgG producing plasma cells in atopic children and the tendency for direct or further class switching to IgE are the seminal events that generate long‐lasting sensitization of mast cells in allergic children. This paper will look at allergy through the role of antibodies other than IgE and give current knowledge of the nature and role of IgG antibodies in the start, maintenance, and resolution of allergy.

## Background

Immunoglobulins of all classes, but especially immunoglobulin G (IgG), are induced as part of the natural exposure to allergens daily. Peanut, milk, and other natural allergens provoke antibody responses, particularly IgG, to incidental ingestion and each person has their own distinct and variable repertoire of antibodies to their diet and environment. Antibody levels to different foods vary greatly in different children despite similar exposure or diet but levels of immunoglobulin G class or subclass antibodies can be associated with levels of allergy and Th2 cytokine responses to sensitising allergens 1. In mice anaphylaxis can be mediated by IgG antibodies, acting through the low‐affinity IgG receptor on macrophages to release PAF (platelet activating factor), inducing smooth muscle contraction and increased vascular permeability in a very similar way to histamine 2. In humans, anaphylaxis has been repeatedly observed in patients with specific IgG but no detectable IgE antibody when treated with variety of intravenous immunoglobulins (for IgA‐deficiency), monoclonal antibodies, dextran, aprotinin, and von Willebrand factor 3. In addition, elevated immunoglobulin G antibodies characterize a number of autoimmune inflammatory syndromes, including rheumatoid arthritis (RA) 4, 5 and systemic lupus erythematosus (SLE) in which the pathology recapitulates the immune damage generated in allergy. In RA, an antibody against the Fc portion of IgG forms the autoantibody or rheumatoid factor (RF) that forms immune complexes that contribute to the disease process 6. Alterations of the T cell receptor ζ chain, with heightened affinity for cell activating IgG1 and IgG3 antibodies, enhance T helper cell activation of B cells and decreased regulatory function in in the pathology of SLE 7. It is thought that approximately a third to half of allergic reactions to foods may involve IgE‐independent mechanisms, such as type II, type III, or type IV hypersensitivity reactions, leading to such allergic symptoms as serum sickness, hives, joint pains, and rashes. Both basophils and mast cells can be triggered by complement though receptors for anaphylatoxins C3a and C5a. Therefore, symptoms of allergy can occur in a range of inflammatory diseases and can be caused by IgG or other IgE‐independent mechanisms.

Sensitization to allergens usually starts in the early maturation of the individual immune system with the development of B cell clones producing specific antibodies of IgE class that continue to trigger reactivity on the surface of mast cells and basophils where they are docked in high affinity receptors 8. Why some children develop IgE to allergenic compounds and others do not and why IgE in some children induces clinical allergy when others have specific IgE without clinical reactivity are still highly controversial. Are IgE producing plasma cells that generate clinical symptoms, such as anaphylaxis or systemic capillary dilation and shock in allergic children, induced in atopic individuals to undergo further class switching by Th2 conditions 9 or is the nature of these allergens such that it triggers development of IgE producing B cells bypassing IgG production? Is there something special about shape or chemical reactivity of molecules such as Ara I in peanuts and Fel d1 in cat dander that induce a B cell development particularly to IgE? Several prominent allergens have enzymatic properties, suggesting that proteolytic enzymes invading parasites secrete that break down connective tissue and allow the parasite access to host tissues, may be particularly active at promoting Th2 responses. Dust mite (*Dermatophagoides pteronyssinus*) Der p 1 is able to cleave CD23 (FcεRII, the low‐affinity IgE receptor) and CD25 (IL‐2 receptor α‐chain) expressed on the surface of some leukocytes 10, leading to dysregulation of the immune response and enhanced production of IgE 11. However, no general homology in small sequences of 10–15 residues across allergen families emerges and no consensus sequence for an allergenic epitope has been found to date 12.

IgM and subsequently IgG are produced predominantly in the first exposure to an allergen. The majority of people have a conventional immunoglobulin reaction to allergenic substances, mainly in the form of Immunoglobulin G antibodies of the five immunoglobulin classes (Table 1), and only a few undergo a process of B cell differentiation that results in the fully class switched plasma cell producing IgE. IgE‐producing plasma cells in peripheral blood are of very low frequency in normal donors (0.06% of all plasma cells) and rise in atopic patients (0.32%) in patients with high IgE levels (average 7.21%) and hyper‐IgE patients (6.54%) 13. The presence of a range of Fcγ receptors on a variety of haematopoietic cells allows IgG subclasses to differentially activate various cells (Table 1) and provoke a range of immune responses.

**Table 1:** Function of immunoglobulins in allergy

Antobody subclasses	IgM	IgD	IgG 1 1,2,3,4	IgA 1,2	IgE
Serum conc. mg/mL (%)	1.5 (8.1)	0.03 (0.2)	13.5 (72.9)	3.5 (18.9)	0.00005 (>0.001)
Complement activation	+++	+++	+	−	−
Placental Transfer	−	−	+	−	−
External Secretions	Maternal milk, nasal secretions	−	Maternal milk, nasal secretions	Maternal milk, nasal secretions, tears, saliva	−
Cellular interaction (Fc receptor)	DC, monocyte (FCmRI)	−	Monocyte, macrophage, DC, B cell, neutrophil, eosinophil, (FcgRI, II, III)	Macrophage, DC, neutrophil, eosinophil (FcaRI)	Mast cell, basophil, DC, monocyte, eosinophil, (FceRI) Bcell, T cell, eosinophil, macrophage (FceRII)

**Figure 1:**
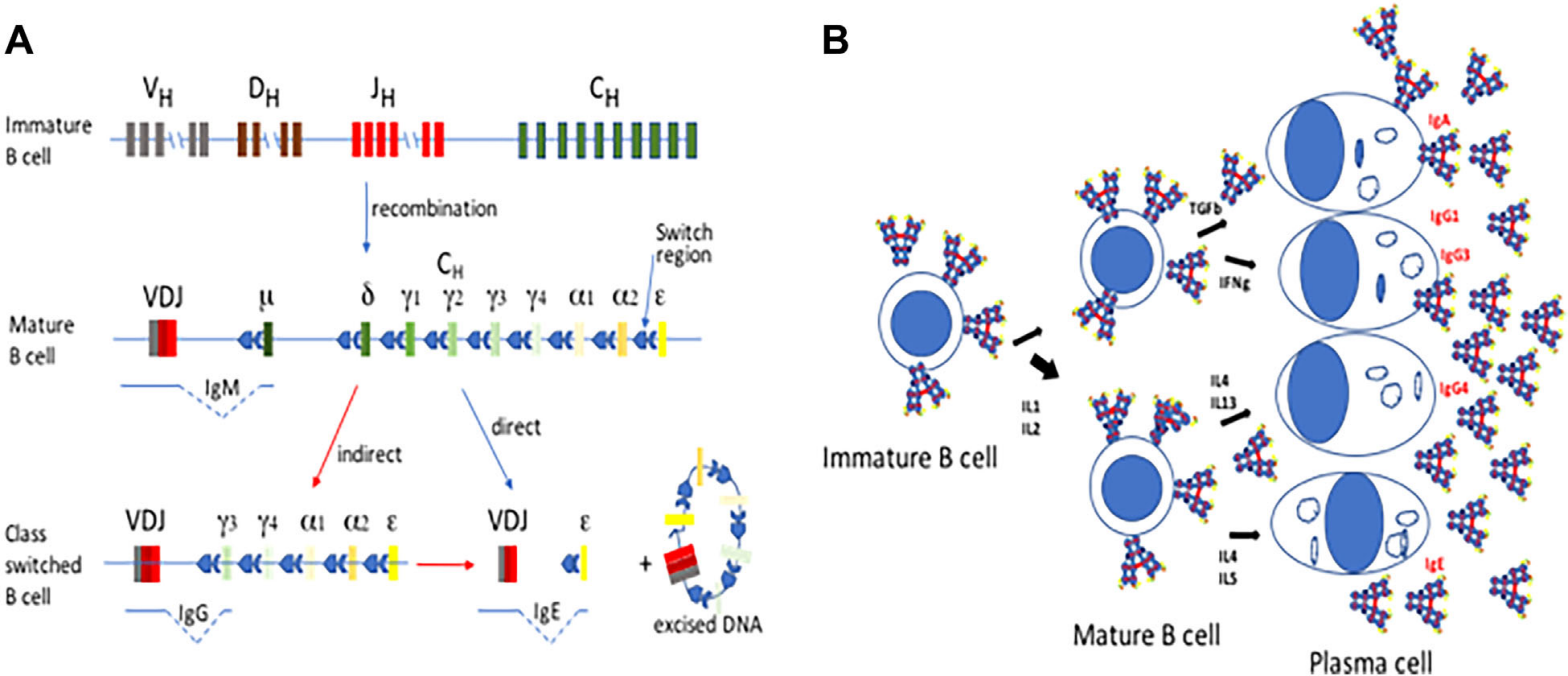
B Cell Maturation and Antibody Class Switching. A: The heavy gene on chromosome 22 consists of many alternative V, D, and J sequences which are deleted except for one sequence each as a loop of excised chromosomal DNA in a process of recombination during B cell maturation in the bone marrow. In the mature B cell the antibody class genes are spliced by switch regions against the chosen VDJ sequence to generate and full antibody protein carried on the surface. In allergy IgE switched B cells can be generated indirectly via IgG producing B cells or switch directly to IgE production. B: Antibody switching in mature B cells is influenced by cytokines produced by T helper cells. IFNγ generates IgG1 and IgG3 secreting plasma cells in the blood while IL4 may generate either IgG4 or IgE producing cells.

Th2 cytokines, such as IL‐4, binding to receptors activate STAT6 (signal transducer and activator of transcription 6), leading to phosphorylation by JAK kinases, homodimerization, and binding to the Iϵ promoter 14, 15 that controls immunoglobulin heavy chain class switching to IgE. LPS stimulation and CD40 signalling by T cells provides other inducible IgE transcription factors, including NF‐κB, and act synergistically to activate transcription of IgE 16. The formation of IgE in B cells can be followed by PCR sequencing of heavy chain genes, with remnants of Sγ1 sequences in the rearranged Sµ‐S heavy gene junctions in IgE producing B cells indicating indirect class switching via preliminary IgG production. It has been shown that mature B cells stimulated with IL‐4 in the presence of anti‐CD40 antibody produce IgG1 for several hours before a switch to IgE while immature, transitional B cells switch preferentially directly to IgE 17. Most IgE switching occurs sequentially from IgG1 or IgG3 production to IgE in a second step 18 which has critical consequences for the affinity of allergen antibodies. Allergen specific B cells in lymph nodes are activated by follicular helper T cells to undergo proliferation during which mutations in the sequence of the variable, antigen‐binding complementarity‐determining regions (CDR, Fig. 1), several per generation, are formed, altering the binding specificity and affinities of lymph node generated antibodies 19. IgE expressing B cells are excluded from germinal centers and do not undergo hypermutation and T cell selection and proliferation 20. B cells that switch indirectly to IgE production would have undergone somatic hypermutation and affinity selection during the IgG stage 21 and generate higher affinity IgE when switched subsequently. The means of exposure to allergen is also important; patients who develop severe allergic reactions to food are often sensitized through damaged skin in early infancy 22. Epidermal dendritic cells (Langerhans cells) thymic stromal lymphopoietin (TSLP) and IL‐33 to food allergens induces Th2 cell‐mediated allergic inflammation in the gastrointestinal tract and the emigration of mast cell progenitors which drive allergic gut reactions through IL‐9 production upon further allergen ingestion 23. So, early class switching to IgE of immature B cells in the respiratory mucosa of children who develop allergic rhinitis and in the GI tract of children who develop food allergy and atopic asthma might be a function of when the B cells are first exposed to the sensitising allergen 24.

Classical allergic sensitization is customarily associated with elevated IgE antibodies generated by class switched B cells in bone marrow and in atopic lymph nodes. Circulating IgE from class switched plasma cells in the bone marrow and blood lodges on high affinity FcϵRI receptors on the surface of basophils and mast cells in tissues, potentiating an immediate hypersensitivity response. Then, contact with the allergen triggers specific IgE lodged on receptors to stimulate mast cell and basophil degranulation and the release of vasoactive amines and other reagents that induce the symptoms of clinical allergy. The IgE‐coated cells are sensitized to the allergen 25 and can generate a clinical response. Most allergens, excepting food allergens, are relatively small, highly soluble proteins that are carried on desiccated particles such as pollen grains or mite feces that diffuse into the mucosa on contact with the airways. It has been estimated that the maximum exposure to the most common pollen allergens in ragweed (*Artemisia artemisiifolia*) airborne pollen concentrations as low as 6–9 grains/m^3^ can produce symptoms of asthma in sensitized children 26. Despite these minute doses, allergic sensitization causing irritating and even life‐threatening Th2‐driven immune responses via specific IgE to these allergens is increasingly prevalent, with rates of allergen sensitisation in children approaching 40–50% globally 27.

A distinct form of IgE receptor, with different affinities and expression patterns on immune cells, contributes to the longevity of clinical allergy. The low‐affinity IgE receptor (FcϵRII; CD23) is expressed on the surface of B cells, as well as other hematopoietic cells, while the high‐affinity IgE receptor (FcϵR1) is expressed on mast cells and basophils (Table 1) as a tetramer (α, β, and 2 γ molecules) and on antigen presenting cells, at much lower levels, as trimers (α and 2 γ molecules). Free IgE has a very short half‐life but once bound to FcϵR1, is expressed for the life of the cell 28. FcϵR1 density of expression on mast cells is proportional to the concentration of elevated free IgE levels and enhanced by IL‐4, C3a, and C5a complement components through C3aR and C5aR (CD88) receptors, nerve growth factor through TRKA receptor, and IgG through FcγR1. Mast cells activated by TLR ligands, such as TLR3 by double‐stranded RNA, induces IFN‐γ production 29, indicating that the mode of mast cell activation can promote or modify allergic sensitization through the high affinity IgE receptor FcϵRI. One of the principal immunological effects of IFNγ is the induction of MHC class II molecules on macrophages. Toll receptor stimulation of mast cells and basophils, principally via TLR2 activation by proteases such as Der p 1 and hookworm antigens, forms the predominant source of IL‐4 in allergen and helminth parasite‐activated PBMCs, suggesting an evolutionary role for basophils and mast cells in antigen presentation in MHC class II molecules and for induction of IL‐4 production and Th2 responses in the immunity to parasite infection 30, 31.

Syndromes that mimic allergic reactions, such as food allergy in atopic dermatitis patients and food protein‐induced enterocolitis syndrome (FPIES) are not dependent on IgE and are generated in the absence of allergen specific and even total IgE. Since basophils express both activating and deactivating Fcγ receptors, including FcγRI and FcγRIIb (Table 2), as well as complement receptors FcγC3α and FcγC5 clinical symptoms can be induced by degranulation caused by IgG antibodies. Thus, IgG antibodies in RA and SLE and diabetes and allergy generate pathology through interaction with mast cells and basophils 32.

**Table 2:** IgG receptor and cellular interaction of IgG subclass antibodies

IgG subclass	**IgG1**	**IgG2**	**IgG3**	**IgG4**								
Serum conc. mg/mL (%)	9 (48.6)	3 (16.2)	1 (5.4)	0.5 (2.7)	IgG receptor function in different cells							
Complement activation	+	+++	−	−	**Mono cyte**	**Macro phage**	MdDC*	Con DC*	p DC*	**Mast cell**	**B cell**	**Receptor action**
FcγR1 (CD64) affinity (k)	600 × 10^5^	−	600 × 10^5^	300 × 10^5^	++	+++	+	+	−	++	−	activation
FcγRIIA (CD32A) affinity (k)	30–50 × 10^5^	1–4 × 10^5^	90 × 10^5^	2 × 10^5^	+++	+++	++	++	++	+++	−	activation
FcγRIIB (CD32B) affinity (k)	1 × 10^5^	0.2 × 10^4^	2 × 10^5^	2 × 10^5^	+	++	+++	+++	+	−	+++	inhibition
FcγRIIC (CD32C) affinity (k)	1 × 10^5^	0.2 × 10^4^	2 × 10^5^	2 × 10^5^	++	+++	+++	−	−	−	−	activation
FcγRIII (CD16) affinity (k)	1–2 × 10^5^	0.3–0.7 × 10^4^	8–10 × 10^5^	2 × 10^5^	++	++	(+)	−	(+)	+/−	−	activation

* mdDC are generated from monocytes in vitro, blood monocytes are divided into CD14+ myeloid DCs (con. DC) and the CD303+ plasmacytoid DCs (pDC). Affinity (k) from Vidarsson et al. 151. Expression from Bruhns and Jonsson 152, 153. +++ high expression, ++ moderate, + low expression, (+) inducible expression, +/− intermittent expression or positive subset.

Evidence indicates that most atopic people start off with a normal broad distribution of food directed IgG secreting B cells but are disposed in certain situations, either by an inherent genetic disposition or experience in the womb or soon after birth to generate a Th2 polarized immune reaction to allergenic compounds, causing a class switch toward mature plasma cells producing IgE. The tendency for the plasma cells to undergo further class switching beyond IgG in atopic individuals and the nature of allergenic epitopes in inducing specific IgE reactions will be established by advances in current immunogenetics techniques in sequencing individual and clonal B cell IGH gene sequences in populations of sensitized and allergic children 33. These technologies will determine how antibody triggering of the B cells that generate allergic symptoms through basophil and mast cell receptors in atopically disposed children occurs and why certain B cells switch to well targeted, high affinity, specific IgE with the same conformational specificity of the germinal center, hypermutated IgG1 or IgG3 antibody to the inducing allergen, causing the sensitization that sets allergic children apart. How this seminal event of sensitizing IgE class switching can be avoided in vulnerable children or reversed by immunotherapy treatment of allergic children may emerge from better knowledge of the events that lead to IgG subclass differentiation.

**Figure 2:**
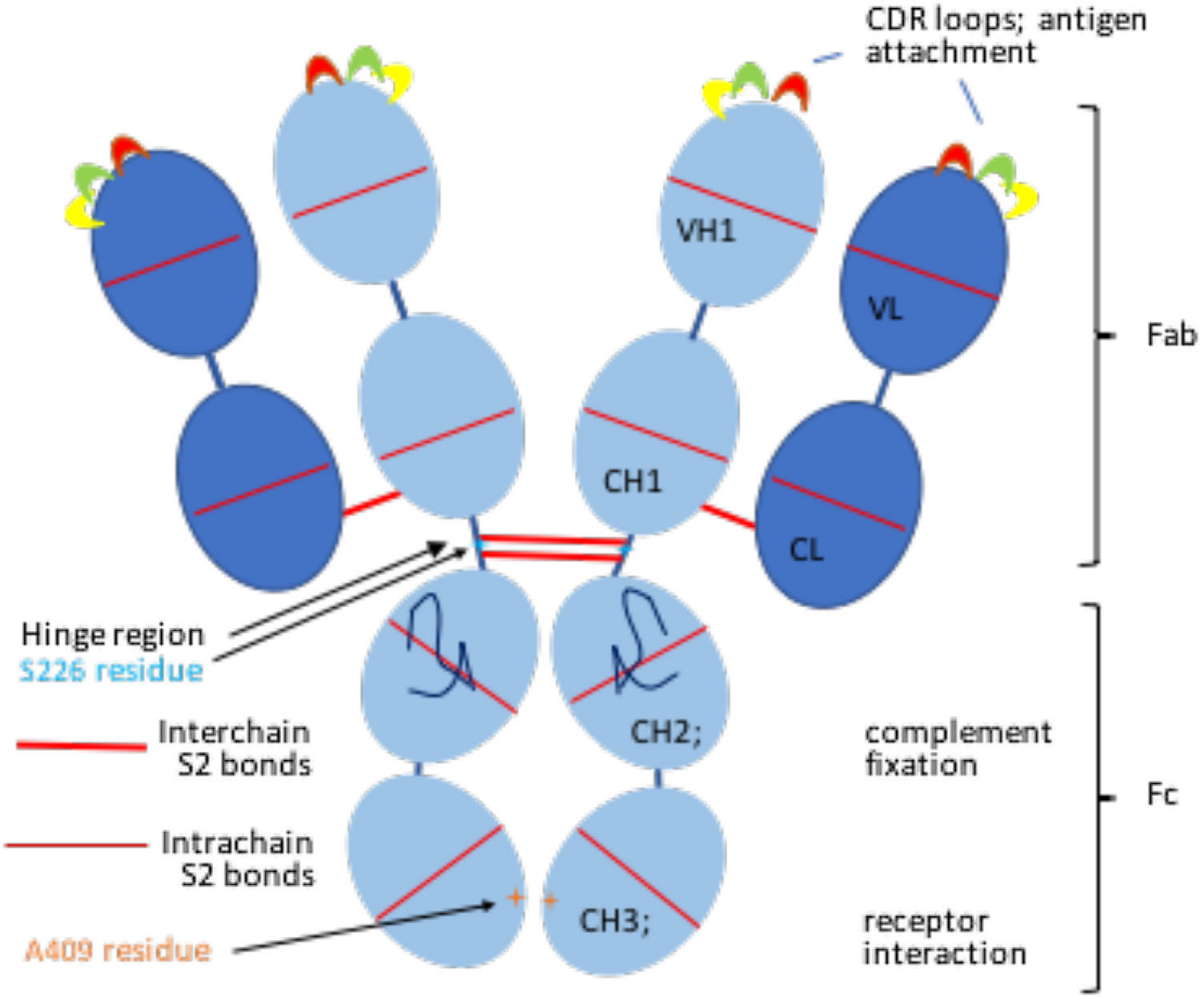
Modular Structure of an IgG antibody. Antibodies are composed of globular domains, 4 per heavy chain, heavy‐chain variable (VH), first, second and third constant (CH1, CH2, and CH3) domains, 2 per light chain, variable (VL) and constant (CL) domains, held together in a strong barrel shape by intrachain disulphide bonds. Light and heavy chains are linked by interchain disulphide bonds. Different domains have different functions, the top domain of both the light and heavy chains carry unique complementarity determining regions (CDR) which project and interact with antigen as loops of unique sequence. Interaction with complement or cellular receptors is the function of CH2 and CH3 domains comprising the Fc portion of antibody. The serine residue at position 228 in the hinge region and the arginine at position 409 within the CH3 domain of IgG4 are indicated.

Finally, as regards the cycle of allergy, it is IgG4, in particular, that is produced or correlates with desensitization of allergy by oral immunotherapy or AIT. IgG4 has an unstable structure and some estimates are that the majority of antigen bound IgG4 is present as monovalent halves of antibody that detach at the intrachain disulphide bond present in the hinge region (Fig. 2). Monovalent IgG4 antigen complexes are unable to bind and crosslink Fcγ receptors and fail to stimulate APC activation but can bind to FcγIIb receptors and induce downregulation of mast cells and monocytes and macrophages 34. Allergy involves several interlocking factors but begins with the production of IgG to allergens during natural exposure and deviates in atopic individuals to form terminally switched B cells capable of induction of an allergic response. Alternative class switching to IgG4 can contribute to the natural amelioration of an allergic response and be provoked in immunotherapy by stimulation of IgG4 production. The resolution of allergy may depend on the generation of IgG4 antibodies that counter sensitising IgE antibodies to allergens.

### Allergic process and the involvement of immunoglobulins

The problem with associating an individual immunological effector in allergy is unequivocally separating the individual actions of the many immune factors in pathogenesis. Human sera contain a mixture of antibodies of different classes and subclasses produced by B cells with a wide range of specificities and clonal sizes. B‐1a cells, resident on pleural and peritoneal surfaces in older mice, generate natural antibodies whereas B‐1b cells in the spleen marginal zone and some mucosal surfaces pick up some microbial polysaccharides, glycolipids, and a few commensal and infectious microorganism antigens 35, including flaggelin, without CD4+ Th cell assistance of any sort. However, these thymus independent antigens are not often associated with allergy. Most well‐known food allergens are presented in the normal fashion by ligation of B‐2 cells when they crosslink surface antibody (IgM or IgD), and are reabsorbed, processed, and re‐presented on the surface in the context of MHC molecules to CD4+ Th cells in germinal centers. The polarization of Th cells in germinal centers in atopic individuals is thought to set off amplification and terminal class switching of further plasma cells to generate allergic responses to allergens. A lack of exposure to microorganisms in infancy in a relatively sterile modern environment is thought to leave modern children with inadequate Th1 or inflammatory challenge and primed to respond in Th2 allergic or anti‐parasitic immune response 36.

B‐1 lymphocytes generate from stem cells in foetal and early neonatal immunity and tend to become localized to peritoneal and pleural cavities in adult mice. These lymphocytes are primarily induced by high molecular weight antigens with low degradability with repeating epitopes, such as bacterial capsular polysaccharides, that elicit rapid antibody responses by multivalent BCR crosslinking in the absence of major histocompatibility complex class II‐restricted T cell help 37. B‐1 cells thus produce natural IgM in the absence of CD4+ cell interaction 38 as a first line of defence against a broad spectrum of infections, such as encapsulated bacteria. B‐2 lymphocytes, on the other hand, emerge from bone marrow later in life, to produce high‐affinity, monospecific class‐switched immunoglobulin that provides long‐lived serological memory. B‐2 cells typically encounter antigen first in the lymph node and proliferate oligoclonally from germinal centers. B‐1 lymphocyte targets, so called T independent antigens, are only very infrequently sensitising allergens as it is thought to be the polarized micro‐conditions in naïve lymph nodes in allergically disposed individuals that cause differentiation of mature B‐2 cells beyond IgG or directly to IgE producing plasma cells. B‐2 cells are selected by the binding of antigen to clonally distributed B cell receptors (BCRs; membrane‐bound antibody), triggering signalling cascades that result in B cell activation 39. Cross‐linked surface antibodies on immature B‐2 cells in nascent germinal centers in lymph nodes and spleen are incorporated when binding multivalent cognate antigen and follicular T cells stimulate B cell amplification and IGH rearrangements on chromosome 22. Primary induction of antibodies including IgE occurs in lymph nodes local to exposure. In rats repeated treated with aerosolized ovalbumin generated specific IgE plasma cells first and foremost in the anterior and posterior mediastinal lymph nodes of the lower respiratory tract 40. IgG, IgA, and IgE are produced locally in humans too as shown by the predominance of specific antibodies to pollen in nasal secretion in hay fever 41. However, follicular T cells (Tfh), a subset of regulatory T cells expressing chemokine receptor CXCR5 as well as costimulatory molecules ICOS and CD40L 42 inhibit switching to IgE and may not be conducive to the formation of allergy 43. Tfh principally produce IL‐21, a cytokine closely related to IL‐2 and IL‐15 which reduced IgE responses and T cell production of Th cytokines to ovalbumin when administered intranasally to mice 44. In IL‐4‐stimulated mouse B cells, IL‐21 down‐regulated IgE production by inhibition of germ line epsilon transcription 45 but when administered to human PBMC stimulated with anti‐CD40 antibody and cultured with IL‐4, IL‐21 enhanced IgE production and the formation of plasma cells 46. Therefore, the restriction of Tfh to the formation of IgE in human does not appear to be as absolute as in mice and a Th2 cytokine microenvironment could potentiate the formation of IgE producing plasma cells in lymph nodes. There are still many uncertainties about the formation and interaction of Tfh and Th cells in lymph nodes in the development of allergy

IgE‐producing plasma cells may have two origins; they may originate from an immature IgM‐producing B lymphocyte transformed directly to IgE secreting plasma cells or from an IgG producing intermediate mature B cell in bone marrow or lymph nodes that has already met its cognate antigen or allergen. Immature lymphocytes triggered by allergens would initially produce weak antibodies with poor affinity in comparison to IgE‐producing cells derived via indirect switching that have undergone affinity maturation as an IgG producing clone. Evidence suggests that most IgE plasma cells are derived from previously antigen‐experienced B cells rather than naive B cells. DNA sequencing of 15,843,270 of immunoglobulin heavy gene rearrangements of B cells from allergic and normal children expressing IgE gave results consistent with indirect switching to IgE from IgG or IgA expressing B cells 33, suggesting that allergen specificity and affinity of IgE in allergy is generated by somatic mutation of preformed immunoglobulin products. Isotype switching frequencies were similar in healthy and allergic subjects which indicates that atopic individuals do not generate higher specific IgE concentrations and wider specificities than non‐atopic children through intrinsic differences in B cells 33.

The class of immunoglobulin produced by B cells is determined by two signals; CD40L ligation provided by CD4 T cells and specific cytokines which activate the promotor of particular immunoglobulin isotypes. Different cytokines direct the differentiation of early B cells and the recombination of the heavy chain class genes on chromosome 22 in particular ways (Fig. 2A). Tfh and perhaps Th cells in local lymph node germinal differentiate when presented with a specific cognate antigen, they secrete cytokines and thereby stimulate a class switch in adjacent B cells. The regulation of IgG subclass switching of murine B cell by CD4+ CD25+ cells T helper cells has been very well studied in mouse models of allergy and it is evident that the ability of specific cytokines to generate specific differentiation is highly controlled in mice (Fig. 2B). IFNγ applied to mouse mixed peripheral blood cells in culture in vitro strongly predisposes to the production of IgG2a antibody producing plasma cells 47, 48. While there is a clear distinction in mice between three lineages of Th cells; Th1 cells producing IFNγ and IL‐2, Th2 cells producing IL‐4 and IL‐5 49, and Th17 cells producing IL‐17 50, IL‐21 and IL‐22, in humans there is some interchange and variation between these cytokine producing Th cells. IFNγ producing Th1 cells also promote IgG1 and IgG3 antibodies in humans and elevated Th2 polarized cells in atopic individuals generate more IgG4 and IgE production 51. But additional discrete T helper cell subsets are involved in human allergy; Th9 cells, induced by TGF‐β and IL‐4, secrete IL‐9 which enhances the growth of mast cells, and can lead to inflammation in the lung and intestines, including intestinal anaphylaxis. IL‐9, in combination with TGF‐β, can induce the development of Th17 cells which are found in the lungs of patients with severe asthma and in the skin of patients with chronic atopic dermatitis. So, in human allergy, while it is probably true that the cytokine microenvironment of the lymph node predominantly affects the activation and shaping of the adaptive immune system, there are influences, including the innate immune system 52 and the degranulation of mast cells and basophils and the activation of eosinophils, that induce an atopic autocrine cycle of cytokine and antibody polarization, leading to higher level of Th2 cells and higher levels of IgE in atopic individuals 53.

Mast cells, the principal effector cell in allergy, release various vasoactive substances, including histamine, SSRA (slow reacting factor of anaphylaxis), and serotonin when triggered by IgE class immunoglobulin to allergens. A large range of pre‐formed immunomodulating molecules such as kinins and proteases are also released from secretory granules. Leukotrienes, prostaglandins, and PAF (platelet activated factor) are synthesized by activated mast cells from arachidonic acid. A whole raft of cytokines (IL‐1, IL‐2, IL‐5, IL‐6, IL‐8, IL‐9, IL‐13, IL‐17, TNFα, and TGF‐β1), chemokines (CCL1, CCL2, CCL3, CCL3L1 CCL4, CCL5, CCL7, CCL8, CCL11, CXCL2) and growth factors (VEGF, PDGF, bFGF, EGF, IGF‐1, and NGF) are synthesized de novo and released soon after activation 54. Besides the dramatic effects of vasodilation in immediate hypersensitivity and anaphylaxis, mast cells have many homeostatic functions, including blood coagulation and flow, smooth‐muscle contraction and intestinal peristalsis, wound healing. The secretion of these factors influences the recruitment, survival, development and function of granulocytes, macrophages and dendritic cells and affects both innate and adaptive immune responses, as well as peripheral tolerance and autoimmunity. By inducing T and B lymphocyte activation, mast cells influence the initiation, magnitude, and maintenance of immune responses and autoimmunity 52. Many of self‐antigens that react with IgE are homologs of environmental antigens, including actin binding protein, profilin, serum albumin, collagen and desmoplakin, and responses to these are thought to result from cross‐reactivity 55. It is possible that inflammation associated with IgE‐dependent mast cell degranulation to environmental allergens induces tissue damage and, in a Th2 dominated immune environment, allows these self‐antigens, no longer sequestered from the immune system, to elicit an IgE response 56.

Immunoglobulin triggered mast cells, through the release of histamine and prostaglandins, also induce the downregulation of IL‐12 and stimulate the production of IL‐10 by dendritic cells, resulting in a decrease in expression of IFNγ and an increase in IL‐4 by T cells 57, 58. IL‐10 also affects B cells, resulting in class switching to IgG4 and suppression of IgE production in peripheral B cells 59. Macrophages may be greater sources of IL10 and can be stimulated to produce IL10 by IgG4 antibody binding to FcγRIIb. IL‐10 production by CD4^+^CD25^+^ allergen‐specific Tr1 (regulatory T) cells, B cells and monocytes induces and correlates with the production of IgG4 and the increase in IgG4/IgE ratio in allergen and peptide immunotherapies in allergy and autoimmune diseases 60, 61, 62 and are thought to underlie the induction of tolerance to allergens 63. However, in allergy, once established, high levels of IgG and IgE producing plasma cells present in peripheral blood of allergic and atopic children persist. The IgE response is further amplified by basophils, mast cells, and eosinophils, which all express FcϵRI, (although eosinophils require activation) and cell‐surface CD40L and secrete IL‐4, thereby driving class switching and IgE production by B cells. Long‐lived IgE response in allergy are not due to IgE memory cells, but are prolonged because plasma cells in the bone marrow can survive for many years 64. The half‐life of mast cell docked IgE on sensitized cells has a much longer duration than free IgG or IgE, resulting in clinical reactivity continuing for the life of antibody sensitized cells.

### Immunoglobulin G and involvement in allergy

Human IgG (Fig. 2) is comprised of four subclasses with different properties and functions as a result of their different heavy chains. The IgG1 constitutes some 65% of the total IgG, with IgG2 forming about 22%, IgG3 about 7%, and IgG4 only about 4% of the total IgG in serum 65. Different functions are conferred by various binding affinities to Fc receptors on different cells (Table 2). All four subclasses of IgG cross to protect the newborn in the first months of life. Placenta cells in contact with maternal blood express FcRn receptors that bind of all four subclasses of IgG molecules equally 66. Bound antibodies are then taken up by receptor‐mediated endocytosis, transported across the cell in vesicles and released into the foetal blood through the basal membrane by polar transcytosis. Infant antibodies in the first 1 or 2 months of life thus follow the serum concentration of maternal IgG antibodies (72% IgG1, 24% IgG3), allowing a period of passive protection for the maturation of immune cells and the amplification of cellular defences before the maternal IgG fades away.

IgG1 and IgG3 antibodies activate complement and clear the majority of pathogens through opsonization and uptake by macrophages, neutrophils, basophils, and monocytes. IgG3 is the strongest complement activator, followed by IgG1 while IgG2 can only activate complement in the presence of high concentrations of antigen and IgG4 does not activate complement at all 67. IgG2 is produced mainly in response to thymus‐independent antigens 68, 69, while IgG4, and IgE, are usually produced only in helminth infection or on exposure to allergens 67.

B cells mature in bone marrow, rearranging the sequences of heavy and light chain genes on chromosomes 22, 2, and 14, respectively, by recombination, splicing together V, D (heavy only), and J segments to form an antibody sequence unique to each B cell by the time it emerges into blood. Cytokines secreted by a T helper cell in antigen presentation binding to the IgD or IgM antibody docked on the surface of a naïve B cell can generate antibody class switching and proliferation of the cognate B cell in an interaction that usually occurs in secondary lymphoid tissue like lymph nodes or spleen. VDJ genes on chromosome 22 are stimulated to recombine with different C regions of the heavy chain gene sequence by switch recombination with deletion of the intervening DNA (Fig. 1A). The mechanism by which mature B cells arrive at the subclass of antibody they secrete (Fig. 1B) is not well determined in humans. The greatest detail about the regulation of IgG subclass switching comes from studies in mice. Murine peripheral blood mononuclear cells (PBMC) provoke IgG2a production by B cells upon the addition of IFNγ to the cell culture 48. Murine inflammatory Th1 cells, CD4 T helper cells secreting IFNγ, generate IgG1 and IgG3, while murine Th2 cells are associated with generation of IgG4 51 but the associations are less direct and only partly understood in humans. IL17, produced by Th17 cells 70 has been associated with IgG1 and IgG3 production by human B cells 71 while human B cells produce IgG4 and IgE when IL4 is added to a PBMC culture in vitro 72, 73, supporting the premise that IgG4 production also occurs in humans by interaction with Th2‐like cells.

The antibody sequence still retains some plasticity in a productive plasma cell. Somatic mutation occurs at a high rate in the mature Ig gene segments of proliferating cells and B cells expressing mutated antibody with improved antigen binding are favored by closer interaction with T cells and higher stimulation through the Fc receptor of the surface antibody. As an immune response progresses, particularly as the free concentration of antigen concentration falls, mutation generates diverse antibodies but those with higher affinity are selected through greater stimulation of the B cells.

Self‐reactive IgG and IgE antibodies are commonly detected in both allergic and autoimmune diseases and contribute to the pathogenesis of atopic dermatitis, RA, systemic lupus erythematosus, Hashimoto's thyroiditis, Graves’ disease, BP and MS, and their animal models 74, 75, 76, 77. In atopy, these autoreactive antibodies are primarily detected in those individuals with severe and chronic diseases such as atopic dermatitis, where cytokine regulation of B cell class switching is less regulated that in patients with more mild respiratory symptoms 74. High affinity receptor for class IgE antibodies on sensitized monocytes, eosinophils, basophils, and mast cells accounts for the prolonged continuation of allergic sensitization, the distinctive symptoms of allergic reaction and much of the long‐term pathology of intermittent allergic exposure. But the presence of both IgG and IgE antibody receptors indicates that both classes of antibodies are involved in the pathogenesis of allergy and common autoimmune diseases and that dysregulation of class switching in a range of autoimmune and atopic diseases generates pathogenic antibodies. Many of the self‐antigens that react with IgE are homologs of environmental antigens, including the actin binding protein, profilin, serum albumin, collagen, and desmoplakin, and responses to these are thought to result from cross‐reactivity. There is speculation that inflammation associated with IgE‐dependent mast cell responses to environmental allergens induces tissue damage and in a Th2 dominated immune environment these self‐antigens, no longer sequestered from the immune system, elicit an IgE response 78.

It may be the very low dose of aerosolized antigens such as pollens and mite antigens that may help polarize the response toward Th2 and the production of IgE. The most efficient antigen‐presenting cells in the respiratory mucosa are myeloid or conventional dendritic cells (con. DC, Table 2) which take up and process the majority of small soluble protein antigens delivered at low dose. Skin borne con. DC migrate to regional lymph nodes and cause differentiation that favors the differentiation of Th2 cells 79.

There is also evidence that the destruction initiated by mast cells and basophils degranulation, possibly by IgG antibodies, generates conditions that favor Th2 cytokine production and class switching to allergen specific IgE. Eosinophil chemotactic factor A released as one of these early phase mediators attracts eosinophils into the area which then release late phase mediators including PGE2 and leukotrienes which generate much more powerful hypersensitivity type I responses, causing an atopic autocrine cycle of cytokine and antibody polarization that influences the IgG subclass toward IgE production 53. Perhaps the polarized immune system of an atopic mother causes polarisation of children in utero, shaping the cytokine induction of B cells in allergen specific germinal centers in the same way as a lack of inflammatory experience in early childhood has the potential to generate a tendency to respond in a Th2 skewed immune response (hygiene hypothesis; Strachan 80). An additional influence in the higher rate of IgE class switching is the repetitive, multi‐epitope nature, the stability, and indigestibility of allergenic proteins that stimulates the prestimulated IgG producing cells into further class‐switching on repeated encounters. Immature B cells may switch more directly from IgM to IgE secretion when exposed to certain allergen conformations in early infancy and the suggestion that early exposure to allergens accounts for higher rates of IgE class switching in atopic and allergic children.

### IgG4 and role in allergy

Human IgG is comprised of four subclasses, each with its own properties and biological functions, as determined by the different heavy chains. IgG1 constituting 65% of the total IgG, IgG2 forming ∼20%, IgG3 some 7%, and IgG4 only about 4% of the total IgG in blood 65. The IgG subclasses differ in their Fc receptor affinity and their ability to activate the complement system. IgG1 and IgG3 antibodies are complement activating and opsonize and cause uptake of invading microorganisms by macrophages by interaction with FcγRI and FcγRIII receptors. IgG3 and IgG1 activate complement strongly, while IgG2 can only activate complement in high antigen concentrations and IgG4 does not activate complement effectively 67. Additionally, there is potential for IgG subclasses to have different roles in immunity, pathology, and allergy by their capacity to bind to different cells via their receptors. IgG4 can bind to Fcγ receptor 1 (CD64) with lesser affinity then IgG1 and IgG4 and does not bind to FcγRII (CD32) or FcγRIII (CD16) 81.

Thymus independent antigens, produced by B1 cells in early foetal in early childhood, generate IgG of subclass IgG2 more than IgG1 or IgG3 antibodies 68, 69. IgG4 antibodies and IgE antibodies are produced to helminth infection but also on exposure to allergens 67. IgG4 production in human, like IgE production is controlled largely by Th2 cells producing IL‐4 and IL‐13 82.

As shown in Table 2, IgG4 levels in the serum of normal individuals are quite low (60 mg/dL). IgG4 does not bind complement but actively inhibits immune precipitation and complement activation by IgG1 antibodies 83 and thereby not only fail to generate significant inflammation but can intervene in inflammatory diseases. The lack of complement and Fcγ receptor binding and the relatively low concentration of IgG4 in serum all suggest that this subclass of immunoglobulin may have regulatory rather than inflammatory or antigen clearance functions. IgG4 production, like IgE, is controlled by T helper 2 cells through IL‐4 and IL‐13 81. IgG4 can bind to Fcγ receptor I (CD64) (though with a lower affinity than IgG1 or IgG3), which presents on monocytes, macrophages, and neutrophils, but not to the other Fcγ receptors (FcγRII/CD32, FcγRIII/CD16) 82. IgG4 does not link with complement and does not bind well to Fcγ receptors of any type. The lack of a conventional IgG activity suggests the role of IgG4 is in immunomodulation. However, elevated IgG4 antibodies are associated with pathology. IgG4‐related disease is a not uncommon systemic, immune‐mediated disease characterized by organ specific IgG4‐bearing plasma cells, mass lesion, or unexplained enlargement, fibrosis, and sclerosis 84, particularly in the pancreas, and the lacrimal, submandibular and parotid glands lymph nodes, thyroid glands and lungs 85. The association of IgG4 with sclerosing diseases may be a bi‐product of excessive anti‐inflammatory cytokines producing expansion of IgG4‐producing plasma cells 86, reflecting dysregulation of T regulatory and Th2 cells rather than the direct causation of fibrosis or sclerosis by IgG4.

Given that IgG4 is the least abundant immunoglobulin, only some 4% approximately of the total IgG found in serum, how is IgG4 able to effect the tollerization of allergens? This may in part be due to the inherent instability of IgG4 in which the interchain disulphide bonds in the hinge region of IgG (Fig. 1) are labile, allowing the molecule to split and exchange monovalent halves. IgG subclasses have more than 95% amino acid sequence identity but a serine residue at position 228 (instead of a proline residue in this position in IgG3) in the core hinge of IgG4 destabilizes disulphide bridges between chains from adjacent cysteine residues, allowing the formation of intrachain rather than interchain disulphides and the separation of IgG4 molecules into two halves 87, 88. Interactions between the CH3 domains of antibody heavy chains in antibody assembly are also critical in the formation of IgG. The CH3‐CH3 interactions of IgG4 are weak, with the arginine at position 409 within the IgG4 CH3 domain of allowing exchange between half molecules 89, 90. It has been estimated that up to 50% of IgG4 may be dissociated, existing as single heavy chain and light chain molecules in serum. These monovalent antibodies do not have the capacity to bind to FC receptors and activate cells but do bind to allergens. Monovalent antibody halves are able to undergo molecular exchange, known as Fab‐arm exchange (FAE), to create antibodies with two specificities 91. New IgG4 molecules with two monovalent specificities are created with less affinity to antigen, giving IgG4 an anti‐inflammatory effect 92. By mopping up antigen but failing to trigger a cellular response, IgG4 could not only deprive IgE antibodies of the allergen to trigger an allergic response, but effectively nullify the action of allergens in triggering cellular activation.

Half molecular exchange (one heavy and one light chain) between two IgG4 molecules, generating new IgG4 antibodies with bivalent reactivity, have been demonstrated in vivo. Van der Neut Kolfschoten et al. 92 estimated that 50% of two IgG4 murine monoclonal antibodies against Fel d1 antigen and Bet v1 antigen underwent Fab arm exchange between when administered to immunodeficent SCID mice. The presence of both κ and λ light chains on 21–33% of IgG4 molecules of five sera tested indicate that Fab exchange occurs in a substantial portion of IgG4 in human serum 93. Bivalent IgG4 molecules have much less affinity to either antigen, reducing inflammation more effectively than clearing antigen 94. Both monovalent IgG4 halves and Fab exchanged IgG4 antibodies, with weak dual specificity, are unable to cross‐link antigens and unable to form large allergen immunocomplexes. Type III hypersensitivity reactions, in which small immune complexes are deposited in tissues and fix complement, giving rise to inflammatory responses and the attraction of leukocytes, constitute a proportion of the pathology of many allergic diseases, such as Farmer's Lung, caused by the inhalation of fungal spores from mouldy hay, Pigeon Fancier's Lung, resulting from powdery pigeon dung, Humidifier Fever, due to protozoans growing in air‐conditioning units, which generate allergic alveolitis. Allergy to penicillin often involves interstitial nephritis, a delayed skin rash, joint swelling, and respiratory distress through type III hypersensitivity when immune complexes are deposited in blood vessel walls and vascular tissues. Antibodies to bee venom in novice beekeepers are predominantly IgG1 that precipitate venom antigen (phospholipase A2). With repeated bee stings, the IgG4 antibody titer rises until it forms more than 90% of the response, at which time immune complexes are not detected 83, demonstrating that IgG4 antibodies are not only non‐precipitating, but also interfere with immune precipitation by IgG1 antibodies. A similar protection against type III hypersensitivity occurs with IgG4 antibodies to Fel d1 in prolonged exposure to cats 95. The low‐affinity Fcγ receptors, FcγRII and FcγRIII (Table 2) are more potently triggered by immune complexes than single IgG molecules 96. This would mean that the weak antigen binding of IgG4 monomers or bivalent FAE molecules would prevent the formation of immune complexes of other IgG subclasses with allergens and thereby reduce engagement of mast cells and other hematopoietic cells in the pathology of allergy.

While immunization typically stimulates IgG1 and IgG2 antibodies, allergy immunotherapy (AIT) is often associated with the production of IgG4. An increase in blocking IgG4 antibodies has repeatedly correlated with the success of the treatment of grass pollen hayfever and the relief of asthma symptoms by immunotherapy 97, 98, 99, 100, 101. IgG4 antibodies have been shown to be effective in preventing even extreme allergic reactions; passive immunization with immunoglobulins from habitual beekeepers (with anti‐venom antibodies almost exclusively of the IgG4 subclass) protected against venom‐induced anaphylaxis 102. The efficacy of IgG4 Fab exchange in vivo has also been demonstrated; IgG4 antibodies to human anti‐acetyl choline receptor (AchR) blocked AChR degradation by IgG1 anti‐AChR‐specific monoclonal antibodies 92. IgG4 antibodies to Fel d1 were strongly correlated with tolerance to both mite and cat allergens while mite IgG1 antibodies associated with asthma 103. Further class switching to IgG4 in allergy is a form of tolerance that has been suggested to explain the observation that domestic animals decrease the risk of asthma in allergic children 103. These observations indicate that development of IgG4 may indeed operate in vivo in allergic children to reduce clinical symptoms of allergy due to reaction with other sensitising allergens.

IgG4 mediated tolerance after AIT or SLIT (sublingual immunotherapy) may be due to its function as a blocking antibody, preventing the allergen causing degranulation of IgE‐bearing mast calls and basophils. AIT also stimulates a complex of B and T cell responses to cope with allergic symptoms: regulatory CD4‐ positive T cells (Tregs) with anti‐inflammatory functions are induced; lymphocytes, monocytes, and dendritic cells secrete anti‐inflammatory IL10 and TGFb cytokines. AIT decreases the production of proinflammatory mediators with reduced migration of mast cells in target organs, IgA antibodies in mucosal surfaces or blood IgG can lead to improvement of symptoms by inhibiting binding of allergen to IgE on mast cells and basophils 103. As IgE‐allergen ligation is also associated with activation of T‐helper (Th2) cells and IL‐4 secretion, IgG4 may also have an effect by modulating this secretion indirectly. IgG4 has been reported to favour tolerance by stimulating IL‐10 production in regulatory T cells 92 as well as B cells 104. When IL10 was measured in after intralymphatic inguinal injections of birch pollen or grass pollen 105, 106 IL‐10 increased in both cases. IL‐10 was shown to reduce proinflammatory cytokine release from mast cells 107. In addition, IL‐10 downregulates eosinophil function and activity and suppresses IL‐5 production by human resting Th0 and Th2 cells 108. IL‐10 also modulates of B cell Ig class switching in favour of IgG4 production 109, generation the increasing IgG4/IgE ratio characteristic of successful AIT treatment and naturally evolved tolerance 110, 111, 112, indicating that the relative abundance of IgG4 is at least one of the seminal events in the resolution of allergy.

**Figure 3:**
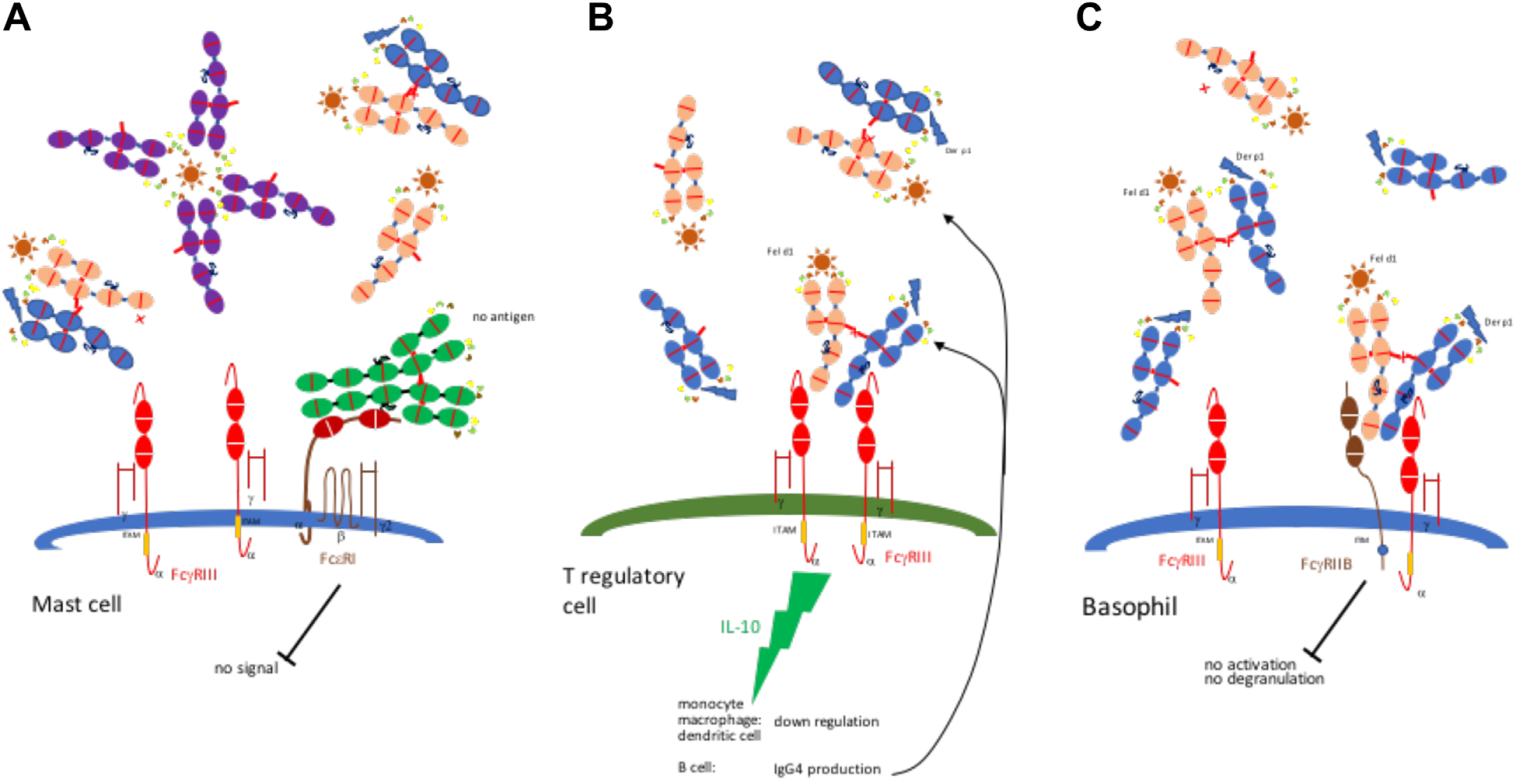
Immunomodulation by IgG4. A: blocking of IgE antibodies. IgG4 antibodies dissociate, mop up free allergen and fail to trigger Fc receptors. B: IL10 production by T regulatory cell, monocytes, macrophages, and DCs induces B cell production of IgG4. C: signaling through inhibitory FCγIIB receptors. ITIM inhibitory stimulus of FCγIIB ligated receptors without crosslinking by half or whole IgG4 prevents mast cell activation through FcγIII. 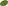 = IgE, 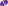 = IgG1, 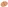 = IgG4 vs Fel d1, 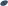 = IgG4 vs Der p1.

Thus, three possibilities may explain the association of IgG4 with favorable AIT and natural resolution of allergy (Fig. 3). IgG4 antibodies may act as blocking antibodies, protecting by blocking IgE‐dependent allergen‐induced activation of mast cells (Fig. 3A). Blocking IgG4 antibodies sequesters allergen so it no longer activates low affinity IgG receptors on mast cells. IgG4 thereby interferes with the IgE‐allergen complex induced activation of Th2 cells, leading to reduced activation of proinflammatory cells. IgG4 production also induces T‐regulatory cells to produce anti‐inflammatory factors like IL10 and TGF‐b which induce an increase in IgG4 production from germinal center B cells. IL10 may be produced in an autocrine fashion from B cells but allergen exposed DC and macrophages and T regulatory cells exposed to IgG4 may contribute a significant proportion when induced through FcγRIII receptors (Fig. 3B). Thirdly, the unique monovalent nature of inhibitory FcγRIIb receptors allows ligation with singe dissociated Fab arms of IgG4‐antigen complexes and overturn the activational stimulation of basophils and B cells due to allergen specific IgG1 and IgG3 antibodies (Fig. 3C). FcγRIIB receptors, however, need to co‐engage with activating FcγRs and immune complexes 113 to inhibit cell activation. Additionally, FcγRIIB receptors have little expression on mast cells and monocytes (Table 2) which may limit the direct inhibitory action of IgG4 to antigen presenting Langerhans and DCs and to preventing degranulation of basophils in blood.

Different effector functions of IgG4 may be involved in different forms of immunotherapy because it addresses a wide range of allergic symptoms from allergic rhinoconjunctivitis to anaphylaxis. The same mechanism cannot be expected for all systems. Anaphylaxis is a rapid process that cannot be prevented through the relatively slow process of T regulatory cell activation but depends on fast‐acting blocking antibodies. In contrast, reducing inflammation in the airways is much more likely to involve regulatory T cells. The significance of regulatory T cells in controlling allergy is highlighted by the fact that Foxp3 (forkhead box P3, a transcriptional regulator that binds to genes in the development and function of regulatory T cells) mutation in mice and humans leads to intense multi‐organ inflammatory responses 85, 114. Development of peripheral T cell tolerance is also characterized by either the deletion or anergy of allergen‐specific T effector cells as well as the generation of allergen‐specific T regulatory cells, together limiting the function of allergen‐specific Th2 cells, mast cells, and other effector cells. This would indicate that different treatments might serve different purposes; treatment with an allergenic peptide might favorably affect specific T‐cell clones but leave clones with reactivity to other allergens unaffected. IL10 directed immunotherapy, on the other hand has the potential to be incompletely allergen specific and favor the resolution of combinations of allergies. The nature of the antigen preparation in AIT is also are important, peptides must be multivalent and resistant to degradation. Denatured allergens or peptides will not be optimally effective in half molecular exchange (one heavy and one light chain) between two antigen bound IgG4 molecules, or generating a new IgG4 with a bivalent reactivity 94 that interferes with IgE cross‐linking of activating Fcγ receptors.

## Conclusions and Therapeutic Applications of IgG4 Antibodies

Human sera contain a mixture of antibodies of different classes and subclasses with a wide range of specificities. Most allergens are internalized by antigen‐presenting cells, including B‐2 cells in germinal centers, macrophages, and dendritic cells, and digested to form peptide fragments expressed on surface MHC class II molecules. CD4+ Th cells differentiate when presented with a cognate antigen, secrete cytokines, and stimulate the class switching in B cells. The IgG subclass generated depends on the type of Th cell response, particularly Th1 cells producing IFNγ and class switching to IgG1 and IgG3 antibodies and Th2 cells producing IL‐4 and IL‐5 and class switching to IgG4 and IgE antibodies 48. Degranulation of mast cells and basophils and the cellular conditions created by hypersensitivity type I responses, and the subsequent activation and degranulation of eosinophils, damage tissue, and influence the IgG subclass toward IgE production, generating an atopic autocrine cycle of cytokine and antibody polarisation 53. The conditions that cause class switching of mature B cells producing IgG1 or IgG3 antibody to allergens in atopic lymph nodes of, children at risk, generating modification of Ig genes on chromosome 22 to IgE production, sensitising mast cells and basophils for degranulation are still under debate. There is evidence that the repetitive, multi‐epitope nature, the stability and indigestibility of allergenic proteins stimulates the preformed IgG producing cells into further class‐switching on repeated encounters. There is also evidence that Immature B cells may switch more directly from IgM to IgE secretion when exposed to certain allergen conformations in early infancy and the suggestion that early exposure to allergens accounts for higher rates of IgE production in atopic and allergic children. Additionally, the genetic influence of an atopic parent may shape the cytokine induction of B cells in allergen specific germinal centers in the same way as a lack of inflammatory experience in early childhood has the potential to generate a tendency to respond in a Th2 skewed immune response 115. Atopic individuals may be predisposed to make Th2 responses and specifically predisposed to respond to some allergens than normal. Studies of atopic families have identified regions on chromosomes 11q, encoding the β subunit of the high‐affinity IgE receptor and 5q whereas there is a cluster of tightly linked genes including those for IL‐3, IL‐4, IL‐5, IL‐9, IL‐12, IL‐13, GM‐CSF, involved in IgE isotype switching, eosinophil survival, and mast‐cell proliferation, that appear to be important in determining atopy. A genetic variant of the IL‐4 gene promoter region that increases expression of reporter gene in experimental systems is associated with raised IgE levels in atopic individuals. Certain HLA class II allele association with allergy, for example, ragweed pollen allergy with MHC II DRB1*1501, imply that presentation of certain allergenic peptides induce stronger Th2 responses. Most atopy associated genetic predispositions are related to the polarization of Th2 cytokine responses to allergens.

Self‐reactive IgG and IgE antibodies are commonly detected in both allergic and autoimmune diseases and contribute to the pathogenesis of BP, RA, SLE, and MS 116. IgG receptor cross‐linking elicits mast cell activation and plays a pathogenic role in allergy and autoimmunity. Many of the self‐antigens that react with IgE are homologs of environmental antigens, including the actin binding protein, serum albumin, collagen, and desmoplakin and are thought to result from cross‐reactivity 74. It is possible that inflammation due to mast cell, basophil and eosinophil degranulation to environmental allergens induces tissue damage and a Th2 dominated immune environment in which these self‐antigens, no longer sequestered from the immune system, elicit an IgE response 78.

Evidently neither the induction, the pathology or the resolution of allergy revolves entirely around specific IgE to allergens. IgG antibodies play a role in the induction of allergic symptoms and pathology of allergy and other autoimmune diseases. IgG antibodies are involved in induction an allergic response, are formulated to bind and eliminate allergenic epitopes, are generated in oral therapy that effectively ameliorates clinical sensitisation and may also be the major means of natural resolution of allergy.

IgG4 may be effective in interrupting the cyclical elaboration of atopic conditions that lead to prolongation of allergic sensitization. IgG4 does not cross‐link complement and does not bind efficiently to activatory Fcγ receptors but, due to the inhibitory action FcγIIb receptors, can interrupt the cyclical induction of Th2 cytokine polarization. IgG4 antibodies may act as blocking antibodies, preventing IgE‐dependent activation of mast cells and interfering with IgG‐allergen complex induced activation of Th2 cells, leading to reduced activation of proinflammatory cells. The IgG4 sequestration of allergen may be more dependent on the weak association of bimodal FAE antibodies as the monomeric form of IgG4 is close to the glomerular filtration threshold (70 kDa) 117 and more prone to degradation by serum proteases, reducing the half‐life (21 h) compared with wild‐type IgG4 (13 days). The lack of inhibitory FcγIIb receptors on mast cells 118, its poor expression on monocytes and neutrophils, may limit the direct inhibitory role of IgG4 on inflammatory cells. However, FcγIIb is highly expressed on circulating B cells and basophils and tissue macrophages and DCs which could denote a role of IgG4 in immunomodulation of systemic allergic reactions and the reduction of allergic responses in lymph nodes.

Given that IgG4 is one of the cardinal signs of developing immune tolerance and may be one of the most effective means by which the immune system generates an escape from the cyclical production of IgE and further Th2 cytokine polarization, how can IgG4 antibodies be used therapeutically? It may be dangerous to promote IgG4 antibodies in all disease. IgG4‐related diseases can affect any organ but are most common in the pancreas, and lacrimal, submandibular, and parotid glands. Patients with an IgG4‐related disease demonstrate high levels of IgG4 in their serum and organ infiltration of IgG4‐bearing plasma cells with consequent fibrosis and sclerosis. In various forms of cancer IgG4 levels correlate positively with T regulatory cells but negatively correlate with cytotoxic T lymphocytes 119 supporting the generation of IgG4 in immune tolerance in cancer. Melanoma can trigger B cell expression of IL‐10 and VEGF, inducing production of IgG4 120. Several carcinomas and cancer cell lines produce IL‐10 and FoxP3, indicating that tumors may promote a biased Th2 response promoting IgG4, but limiting immune responses and enabling the escape from immune clearance. Therefore, elevated serum IgG4 levels have been associated with poorer prognosis in biliary tract cancers 121 and in malignant melanoma 120, 122. Preliminary clinical trials have disclosed adverse reactions with some formulations of IgG4 antibodies. For example, some volunteers involved in the IgG4 anti‐CD28 clinical trial with antibody TGN‐41264 experienced a “cytokine storm” lead to hospitalization with to multiple organ dysfunction, possibly due to the binding of IgG4 immunocomplexes with FcγRIIA and FcγRIII receptors on T helper cells 123.

Nevertheless, the deficiency in triggering of many cellular effector functions makes IgG4 an attractive therapeutic monoclonal antibody format. Pembrolizumab and Nivolumab, for example, both anti‐PD‐1 (programmed death‐1) IgG4 monoclonals in the USA for treatment of melanoma since 2014, block the ligation of the immunoinhibitory PD‐1 receptor on T‐cells, but do not elicit ADCC or complement‐dependent cytotoxicity. Antibodies that target PD‐1 and CTLA‐4 in cancer therapy are thought to act directly by antagonising these inhibitory receptors, and there is no significant evidence to indicate that engagement of Fcγ receptors plays any important role in their therapeutic activity. As the principal inhibitory receptor targets of cancer immunotherapy are expressed either on T cells or on antigen presenting cells, IgG4 has been the isotype of choice in the formation of monoclonal antibody therapeutic reagents as IgG4 does not cause the depletion of cytotoxic T cells or APCs through Fc mediated mechanisms. Therefore, Nivolumab and Pembrolizumab which are in use and MDX‐1105 and BMS‐663513 which target PD‐L1 and CD137, respectively, may have longer duration in serum owing to their IgG4 isotype.

Based on the observation that IgG4 antibodies exchange their Fab arms through a dynamic process that involves separation of the two heavy chains and reassembly into the full molecule, conditions were adapted to generate stable bispecific IgG1 molecules by controlled FAE 124. The suitability of this process for commercial‐scale manufacturing was demonstrated by production of a bispecific IgG against EGFR and CD20 (DuoBody) under controlled reducing conditions, with more than 95% Fab arm exchange on a kg scale 125. However, both Pembrolizumab and Nivolumab have been generated with a (IgG3) proline residue at position 228 in their hinge regions to prevent dissociation. Several IgG4 antibodies currently in clinical trials, with wild‐type or stabilized hinges, are directed against cytokines to alleviate the symptoms of allergy or inflammation, including Reslizumab and Tralokinumab for the treatment of asthma 126, 127 and Ixekizumab for the treatment of psoriasis 128. Reslizumab, approved in 2016, binds to IL‐5 with high affinity, inhibiting IL‐5 signalling and reducing the production and survival of eosinophils. Tralokinumab neutralizes IL‐13, a key respiratory cytokine that drives inflammation, airway hyper‐responsiveness, and excessive mucus production in impaired lung function, contributing to the severity and frequency of asthma attacks. Ixekizumab neutralizes IL‐17, a cytokine directly activates keratinocyte genes including those for beta‐defensins, antimicrobial peptides (AMPs), and chemokines and synergistically interacts with TNFa to generate psoriatic skin lesions. These IgG4 antibodies act directly to neutralize and antagonize the binding of the cytokines so that the involvement of Fab antibody exchange in the action of these monoclonal antibodies (mAb) is unlikely. Similarly, Dupilumab, an anti‐IL‐4Rα IgG4 monoclonal antibody undergoing review evaluation for atopic dermatitis, Galcanezumab an IgG4 antibody targeting calcitonin gene‐related peptide, being studied for use in patients with severe migraines, and Fasinumab, an IgG4 mAb targeting nerve growth factor 129, being evaluated in phase 3 clinical studies as a treatment for osteoarthritis pain, owe their probably action to direct interference with the action of the target protein and not immunomodulation.

Similarly, Ibalizumab and PRO140, both new IgG4 mAb undergoing evaluation as treatments for human immunodeficiency virus (HIV)‐1 infection, act directly on viral receptors on cells and owe their efficacy to interfering with virus binding without disturbing the cells’ immunological functions. Ibalizumab binds to the second extracellular domain of CD4 130, while PRO140 inhibits R5 CCR5‐tropic HIV by targeting the chemokine receptor CCR5 131, which acts as a co‐receptor to viral entry of macrophages, dendritic cells and memory T cells. GNbAC1, an IgG4 mAb currently in stage II clinical trials for safety and pharmacokinetics analysis, neutralize the expression of the envelope protein of Mulitple Sclerosis Retrovirus 132, an endogenous human retrovirus that has been linked with MS. Thus, the unique capacity of IgG4 to form bivalent antibodies in vivo through FAE or to block cell activation by weak Fc occupation has not yet been exploited in the formulation of monoclonal therapeutics.

Instead bivalent antibodies, sometimes involving IgG4, are being formulated for a new generation of wide‐ranging therapeutics. A bivalent IgG4 antibody with arms specific to factor IXa and factor X, termed hBS23, binds and brings these two factors into juxtaposition, mimicking the cofactor function of factor VIII to alleviating bleeding in hemophilia A 133. Due to the deregulation of multiple factors in many diseases, bispecific antibodies engaging two targets offer greater potential for therapeutic efficacy as well as overcoming escape mechanisms observed in therapy of single targets. The IgG4 monoclonal antibody Ibalizumab was paired with half antibody anti‐CD70 molecules by site‐directed mutagenesis 134 to create hybrid bivalent antibody with preferential binding and selective depletion of CD4+/CD70+ T cells. A bispecific mAb that neutralizes both IL‐4 and IL‐13 for the treatment of asthma, based on the IgG4 antibody Lebrikizumab, generates higher titers than its predecessor, an IgG1 bispecific antibody 135.

At least twelve bispecific mAbs are currently being evaluated in clinical studies 136 and liable to be available as therapeutics in the near future. With many methods developed for conjugating monovalent antibody halves, few of these mAbs involve IgG4 and few are directed against allergy or are designed to block inflammation. The monoclonal antibody treatment of allergy began with the licensing of Omalizumab, an IgG1 mAb that binds specifically to the CH3 domain of IgE, blocking its interaction with FcϵRI on mast calls, basophils, and other cells 137, 138. Administration of Omalizumab reduces FcϵRI receptor density on and the influx of cells involved in allergic responses and, when as a treatment for asthma, improved lung function and quality of life, and reduced the need for corticosteroids 139. Lowering free IgE levels may downregulate the levels of IgE receptor expression density on the surface of mast cells in time, but the ability of Omalizumab to cause the dissociation of bound IgE is more likely to explain the rapid and prolonged intervention in chronic urticaria 140. The involvement of IgE in other diseases has started the assessment of Omalizumab in allergic rhinitis, atopic dermatitis, food allergies, mastocytosis, and eosinophilic gastrointestinal disease 141, 142. However, there are serious drawbacks to the use of Omalizumab; it costs $10–30,000 annually to treat an individual but moreover it can cause anaphylaxis and fatal autoimmune reactions at a rate of 1–2 per thousand, prompting the FDA to have a black‐box warning placed on Omalizumab 143.

Other mAbs have also been tested in allergic disorders, including agents Th2 and Th2‐promoting cytokines, IL‐4, IL‐5, IL‐9, IL‐13, IL‐31, and thymic stromal lymphopoietin (TSLP) 144. Dupilumab, a human anti‐IL‐4 α receptor mAb (IgG4) was shown to reduce asthma exacerbations, improve lung functions and reduce Th2‐associated inflammatory markers in patients with persistent, moderate‐to‐severe asthma 145. In AD patients, dupilumab decreased expression of genes upregulated in AD lesions by 26% 146 and four further clinical studies in atopic dermatitis, and in asthma, are in process. Anti‐IL‐5 neutralizing mAbs Mepolizumab and Reslizumab and Benralizumab that blocks the IL‐5 α receptor have been developed 147. Reslizumab, targeting IL‐5, a cytokine involved in the maturation, recruitment, and activation of eosinophils, improved lung function, asthma symptoms and quality of life in two Phase 3 studies, without significant side effects 148. TSLP is an epithelial cytokine strongly associated with symptoms and severity of the with asthma and AD 149. mAb AMG 157, that prevents TSLP receptor binding, reduced bronchoconstriction, eosinophil numbers, and indexes of airway inflammation and further trials for severe asthma and AD are underway. Preliminary investigations of mAbs to IL‐9, IL‐13, IL‐17, and IL‐4/IL‐13 are ongoing but optimized formulations of mAb therapy for allergy or asthma are still several years away from clinical use 150. The different properties of IgG isotypes, resulting from differences in binding to serum complement proteins and Fcγ receptors on immune effector cells will continue to be utilized in formulating antibody therapies specific to various targets in allergy. The engagement of IgG1 isotype antibodies of complement proteins and to trigger antibody‐dependent cellular cytotoxicity and the ability of IgG4 isotype antibodies to block Fcγ receptors on immune cells could provide a broad range of therapeutic antibodies with differing effector functions to overcome the lack of improvements with conventional therapy. The use of mAbs that target inhibitory signals rather than blocking activating could be an additional strategy to explore in overcoming allergic responses.

## Ethical Statement

No human or animal subject was involved in this study, no ethical approval was required for the manuscript.
